# Computed tomography-guided preoperative semi-rigid hook-wire localization of small pulmonary nodules: 74 cases report

**DOI:** 10.1186/s13019-019-0958-z

**Published:** 2019-08-19

**Authors:** Guang Zhao, Xiuyi Yu, Weiqiang Chen, Guojun Geng, Ning Li, Hongming Liu, Pan Yin, Long Sun, Jie Jiang

**Affiliations:** 1 Department of Thoracic Surgery, The First Affiliated Hospital of Xiamen University, 55 Zhenhai Rd., Xiamen, 361003 China; 2 Department of Nuclear Medicine, The First Affiliated Hospital of Xiamen University, Xiamen, China

**Keywords:** Hook wire, Video-assisted thoracoscopic surgery, Localization, Small pulmonary nodule

## Abstract

**Objectives:**

The study aimed to retrospectively evaluate the success rate, utility, practicality and results of pre-operative CT (computed tomography)–guided semi-rigid single hook-wire placement and the pathology results of small pulmonary nodules (SPN).

**Materials and methods:**

Seventy-four patients with 81 small pulmonary nodules underwent CT-guided semi-rigid single hook wire localization consecutively between 2016 and 2017 were reviewed. VATS (video-assisted thoracoscopic surgery) resection of lung tissue containing each pulmonary nodule and were performed in the direction of hook wire. The success rate and utility of the localization, hook wire related complications, the histopathology of SPN are analyzed.

**Results:**

The semi-rigid hook wire was performed successfully in all 81 small pulmonary nodules within mean time of 10 min (8–13 min, SD: 1.58 min). Compared with solid nodules, GGOs (ground-glass opacity) were more frequently malignant (*p* < 0.05), with an OR (odds ratio) 8.59 (95%CI, 0.967, 412.845). Of the pure GGOs, 9 (25%) nodules were classified as AIS, 10 (27.8%) nodules were classified as MIA and 22 (57.9%) of the mGGOs were lung cancer. According to multivariate analysis, the malignant hazard was as high as 6.533-fold higher in nodules with a size larger than 10 mm compared with those smaller than 10 mm. GGOs with tiny blood vessels showed a statistically significant correlation with malignancy. Surprisingly, no statistically significant difference in the incidence of lung cancer in age. No major complication occurred.

**Conclusions:**

Preoperative localization of small pulmonary nodules using semi-rigid single hook wire was found to be practical and safe, which allows for proper diagnosis. Incidental small pulmonary nodule, especially GGO larger than 10 mm needs to be taken seriously.

## Introduction

Despite the fact that the death rate of lung cancer declined 38% between 1990 and 2012 among males and 13% between 2002 and 2012 among females [1], lung cancer remains as the most common incident cancer and is the leading cause of deaths among cancer patients around the world, including China [2, 3]. Due to the lack of clinical symptoms in early stage, most lung cancer has been diagnosed at a distant stage, with an only 17% 5-year survival rate [4].

Low dose spiral CT (computed tomography) screening is emerging as a promising strategy in improving lung cancer survival rates due to its advantage in earlier detection. Results from the National Lung Cancer Screening Test (NLST), a randomized clinical trial of more than 50,000 people, showed that screening with low-dose CT could reduce lung cancer mortality by 20% compared to the standard chest x-ray [5]. Benefit from the widespread use of LDCT, a great increasing number of small and non-determined pulmonary nodules have been detected in its early stage**,** especially ground glass opacities (GGO). A small pulmonary nodule was defined as nonsolid (pure GGO) when the underlying parenchyma was visible and there were no solid components except for branching blood vessels within the nodule, and there is no atelectasis, hilar swelling and pleural effusion in the nodule. Nonsolid nodules may develop into an internal solid component and thus may change from being nonsolid to becoming part-solid, which refers to mixed GGO [6]. It can be a manifestation of inflammation, infection, fibrosis or other benign lesions, but it can also be adenocarcinoma in situ, adenocarcinoma or a precursor of adenocarcinoma (atypical adenomatous hyperplasia). As imaging techniques are improving and more nodules are incidentally detected, optimal management of SPN becomes an urgent need for SPN. Study [7] has shown that the risk of malignancy was higher in nodules that measured between 5 and 10 mm (range, 6 to 28%), and it was very high in nodules with the size larger than 20 mm in diameter (range, 64 to 82%). Two studies [8, 9] found pure GGOs to be predominantly malignant (59 to 73%). Thus, a more reliable diagnosis and treatment of uncertain pulmonary nodules is still needed.

Video-assisted thoracoscopic surgery (VATS) has been widely used for small pulmonary small nodules (SSPN), as it represents a minimally invasive way for definitive resection, and it also provides increased comfort for the patient and lower morbidity compared with standard thoracotomy procedures. CT-guided percutaneous needle aspiration biopsy (NAB) of the lungs is widely used to diagnose pulmonary lesions, whereas the diagnostic yield of CT-guided NAB for GGO lesions has been reported to be significantly lower than that for solid lesions because of the fewer cellularity in GGO [10]. Most small pulmonary nodules (especially GGO) were hard to palpate during VATS due to their size and special texture that similar to lung parenchyma [11]. Despite the fact that there are many techniques for preoperative localization of pulmonary nodules (intraoperative ultrasonography, lipiodol, contrast media, dyes, microcoil, finger palpation, radio-guided), their inevitable disadvantages still limit their wide application [12].

We previously conducted a localization experiment in the swine lung and reported that among the commonly used three location methods, semi-rigid hook wire showed better operability and practicability than double-thorn hook wire and micro-coil [13]. In the present study, we continued to use the semi-rigid single hook wire localization technique for individuals during VATS resection. We evaluated the success rate and safety of the semi-rigid single hook wire system and explored the correlation of clinical and radiologic characteristics and histopathologic features about small pulmonary nodules.

## Materials and methods

Our institutional review board approved the retrospective study and waived the requirement for informed consent for the use of the patients’ medical data. An informed consent was obtained from all patients before semi-rigid single hook wire placement.

### Patients

Between April 2016 and August 2017, 81 preoperative localization procedures using a semi-rigid single hook wire were performed in 74 patients at our institution. All nodules were examined under CT, and then VATS was considered for diagnosis and (or) treatment. Preoperative CT-guided hook wire localization was indicated when thoracic surgeons considered that the SPN would be difficult to palpate during VATS. After explaining the necessity and risk of the procedure, a written informed consent was obtained from all patients. Selection for preoperative localization was based on at least one of the following CT findings: small nodules less than 10 mm, located at distance of > 10 mm from the visceral pleura, a predominant ground-glass component in the nodule. Patients’ characteristics are shown on Table 1. Whether to perform a surgery is mainly based on the National Comprehensive Cancer Network pulmonary nodules guidelines.

**Table 1:** Demographic features of patients and radiological features of nodules

Patients	74
Location procedure	81
Sex ratio(men/women)	32/42
Mean age (y)	53.5 range (24–72)
Tumor history	none
Location	
Upper lobe	48 (59.3%)
Middle lobe	4 (4.9%)
Lower lobe	29 (35.8%)
Right lung	52(64.2%)
Left lung	29 (35.8%)
Aspect of the lesion	
Pure GGO	36 (44.4%)
Part-solid	38 (46.9%)
Solid	7 (8.7%)
Number of hook wires used (single/double)	74/7
Nodule size (mm) (MEAN ± SD)	8 ± 3.46
Distance from lesion to pleural surface (mm) (MEAN ± SD)	7.6 ± 7.79
Procedure time of hook wire localization (min) (MEAN ± SD)	10 ± 1.58

### Procedure for hook wire localization

**Fig. 1:**
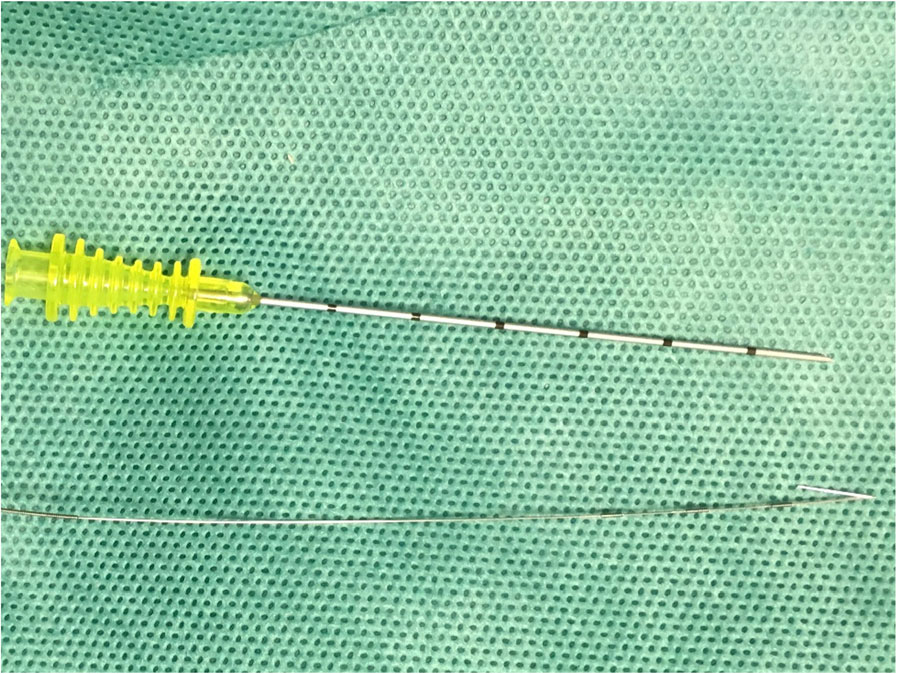
Device: insertion needle and semi-rigid single hook wire

**Fig. 2:**
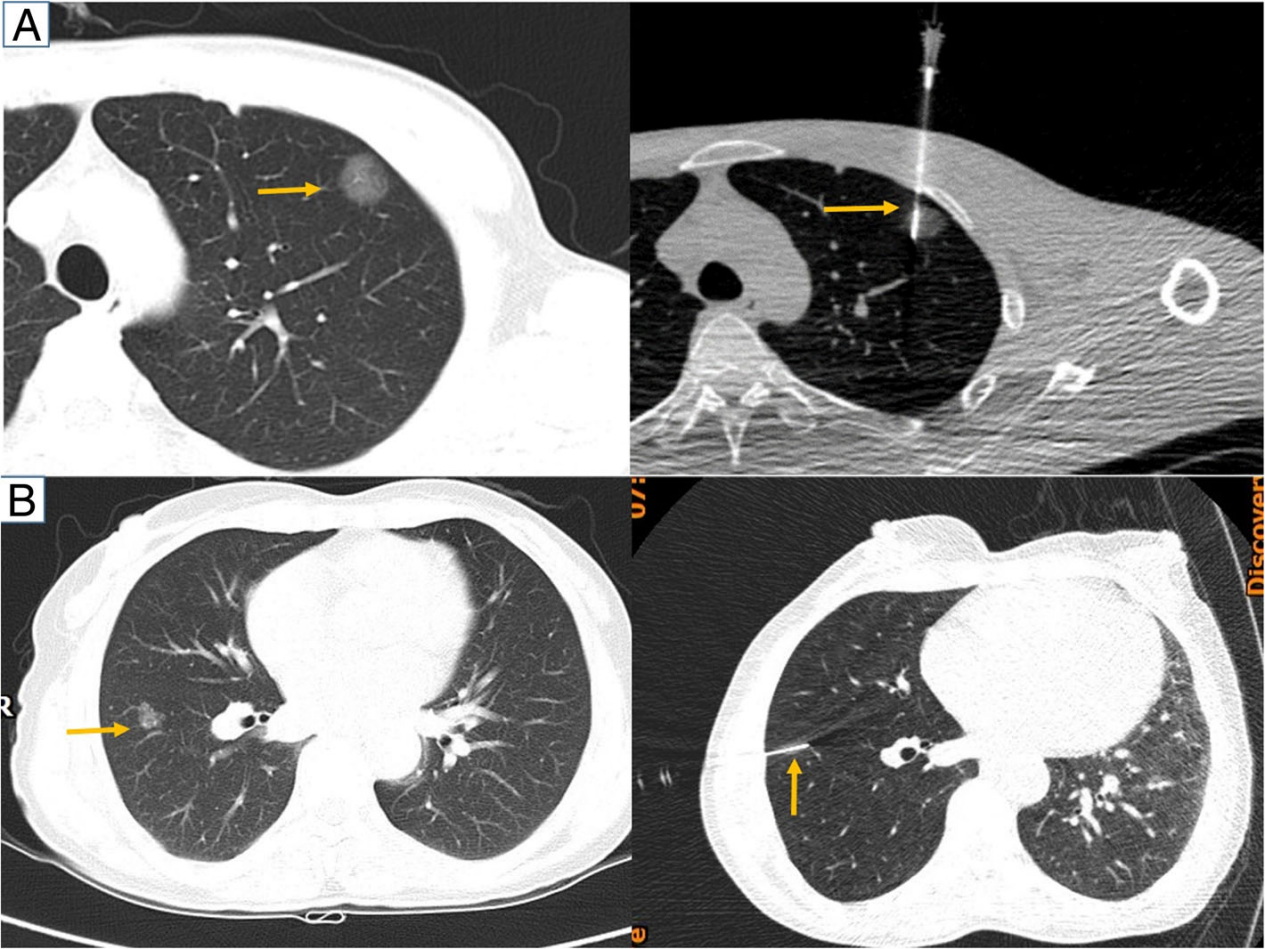
Computed tomography-guided semi-rigid single hook wire localization. **a**, **b** An initial CT was performed to determine the puncture site. After the localizing needle was inserted into the lung, a CT scan was obtained for confirmation (Yellow arrows). **b** The wire was located 2.5mm higher than the nodule

The CT-guided semi-rigid single hook wire was performed on the day of surgery. Procedure was performed under local anesthesia by an experienced radiologist and a thoracic surgeon. A spiral-CT of the partial region was performed with 5-mm slice thickness, with the patient in a supine or prone position, which depending on the site of the lesions. Three metallic grid pattern was laid above the insertion region of the interest. After sterilization of the skin around the puncture site and local anesthesia, a single semi-rigid hook wire (20-gauge needle GHIATAS® Beaded Breast Localization Wire, 7 cm of needle length) (Fig. 1) was gradually inserted into the pleura with sequential CT guidance (Fig. 2), with the patient in breath-hold after deep breathing. Subsequent CT scan was performed to confirm the 20-gauge needle inserted and advanced close to the nodule, but not into it (avoid the tumor disseminating) (Fig. 2). The horn of hook wire would be released and anchored the lung parenchyma around the nodule after the cannula was withdrawn. CT examination was performed immediately after placement to check for if procedure-related pneumothorax and hemorrhage happened. After localization, semi-rigid wire outside the skin surface was cut to about 1 cm and covered with sterile gauze. Immediately after the procedure, patients were transferred to the operating room as soon as possible and VATS was started within 1 h. The color of the nodule’s section were also observed after resection.

### Thoracoscopic surgery

**Fig. 3:**
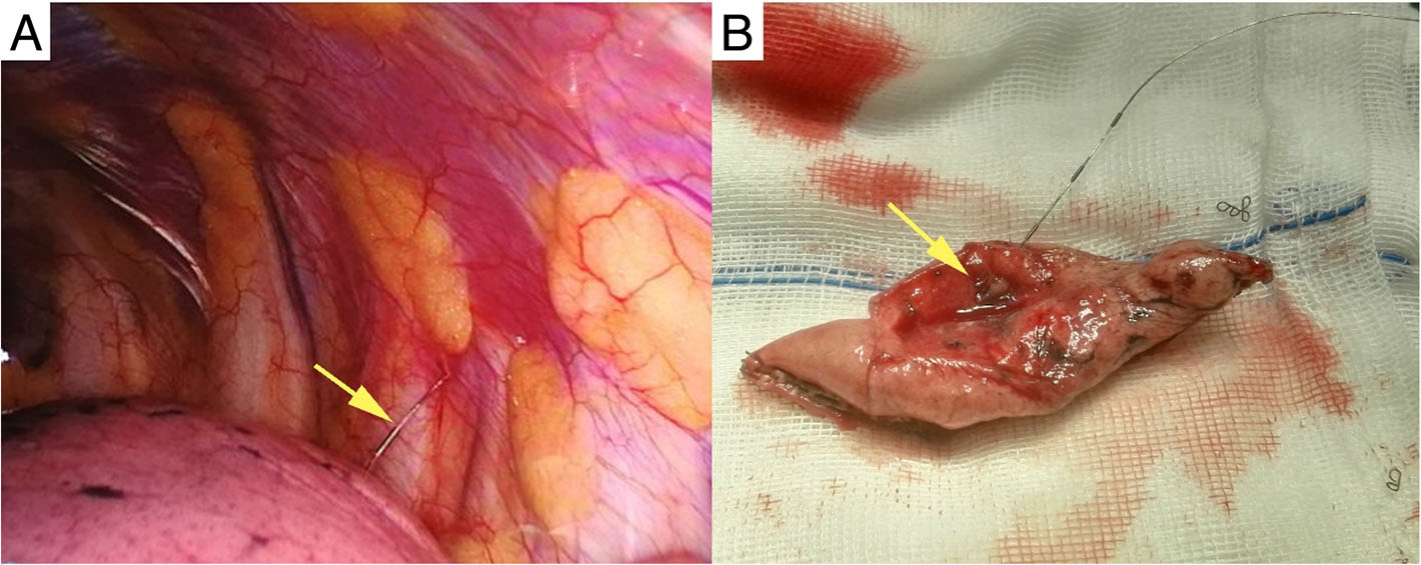
**a** Placed semi-rigid hook wire during operation. **b** Cut the SPN according to the guided wire after pulmonary wedge resection

Thoracoscopic surgery was performed under routine double-lumen trachea cannulation and one-lung ventilation along with general anesthesia. The observational incision was located at the 7th intercostal space on the mid axillary line, the main operating incision was located at the 4th intercostal space on the anterior axillary line, and the auxiliary operating incision was located at the 7th intercostal space on the posterior axillary line. Explorative thoracoscopy was performed and the guide-wire was carefully lifted, after which the nodule was resected with endostapler (45 mm, ENDO-GIA, Ethicon, Hamburg, Germany). The resected tissue was taken into a surgical bag (to avoid the possibility of tumor implantation) and removed out from the pleural cavity. The specimen was histologically examined by frozen-section examination during surgery. Surgical margins were also histologically examined at surgeons’ discretion based on macroscopic status of specimen. If the nodule is diagnosed as benign, metastatic tumor or adenocarcinoma in situ, additional resection would be unnecessary. If frozen section examination revealed primary adenocarcinoma, lobectomy and mediastinal lymph node dissection will be conducted [14]. VATS wedge resection and nodule are shown on Fig. 3. All possible factors including clinical and radiologic characteristics were performed multivariate analysis by logistic regression to determine the risk of malignancy. In the present study, the colors of the nodule were also recorded. The duration of the CT-guided hook guide-wire localization was also calculated.

### Statistical analysis

IBM SPSS Statistics, version 19.0, (SPSS Inc., Chicago, IL) was used for statistical analysis. A *p* value< 0.05 was considered to indicate a statistically significant difference. Multivariate analysis was performed by logistic regression to determine the risk of malignancy based on the radio-logical fiand patients clinical features. The descriptive statistics were presented as mean ± SD.

## Results

Totally 74 consecutive patients with 81 nodules (32 men and 42 women) were involved, with their ages ranging from 24 to 72 years old (mean age 53.5 years). The semi-rigid hook wires were all successfully placed guided by CT scan. All the 81 pulmonary nodules were adequately resected through VATS with histologically free operative margins of the specimens. Seven patients underwent 2 locations simultaneously because of 2 nodules in the same lung. Mean pulmonary nodule size was 8 mm (range 2.4–17.1 mm) and the mean lesion distance to the pleural surface was 7.6 mm (range 0–31.8 mm). The semi-rigid hooked guide-wire was positioned successfully in all nodules within mean time of 10 min (8–13 min, SD: 1.58). Of the 81 pulmonary nodules, 48 nodules (59.3%) located in upper lobes, 4 nodules located in middle lobes and 29 nodules (35.8%) located in lower lobes. There were 52 nodules (64.2%) located in right lung and 29 nodules (35.8%) located in left lung respectively. Of the 46 malignant nodules, 19 (41.3%) were in left lung, 26 (56.5%) were in right lung and the number of malignant tumors in upper, middle and lower lobes were 30 (65.2%), 1 (2.2%) and 15 (33.3%) respectively. In solid nodules, only one nodule presented as malignant (invasive adenocarcinoma), with a diameter 11.4 mm, locate in right upper lung. Histological consequence revealed AIS of 22 nodules (27.2%), MIA or invasive of 24 nodules (29.6%), Fibrosis of lung tissue hyperplasia of 9 (11.1%) nodules, lymph node hyperplasia of 5 (6.2%), interstitial fibrosis of 1 (1.2%), and granuloma of 5 (6.2%).

Table 2 summarizes the results of the histological examination of the excised SPN. Out of all 81 small pulmonary nodules, 46 were malignant and 35 were benign (include 8 AAH--atypical adenomatous hyperplasia). The number of malignant lesions in pGGOs, mGGOs and solid nodules were 22(61.1%), 23 (60.5%) and 1 (14.3%) respectively.

**Table 2:** Complications and histological results

Type of complication	Number of procedure
Asymptomatic pneumothorax	1 (1.25%)
Parenchymal bleeding	1 (1.25%)
Dislodgement	2 (2.5%)
Conversion to thoracotomy(rate)	0 (0%)
Mean procedure time(minutes)	10 (8–13)
Histological findings	
Malignant lesion	46 (56.8%)
Micro invasive or invasive Adenocarcinoma	20 + 4 (29.6%)
Carcinoma in situ	22 (27.2%)
AAH	8 (9.9%)
Benign lesion	27 (33.3%)
Lymph node hyperplasia	5 (6.2%)
Granuloma	5 (6.2%)
Fibrosis of lung tissue hyperplasia	9 (11.1%)
Pulmonary fungus	2 (2.5%)
Collagen nodules	4 (4.9%)
Interstitial pneumonia	1 (1.2%)
Bronchial leiomyoma hyperplasia	1 (1.2%)

According to multivariate analysis (Table 3), we finally identified four pre-operative clinical and radiological factors related to malignancy (Table 3): gender as female (OR: 2.825); maximum diameter of the nodule more than 10 mm (OR: 6.533); radiological performance: GGO VS solid (OR:8.590) and GGO with blood vessels (OR: 5.322).

**Table 3:** Results of the Multivariate Logistic Regression Analysis

Variable	*p* value	OR (95% CI)
Woman/men	0.035	2.825 (0.995,8.338)
≥55 Year-old	0.150	2.172 (0.761,6.430)
Upper lobe/middle or lower lobe	0.485	1.418 (0.506,3.990)
Diameter (≥ 10 mm/<10mm)	0.004	6.533 (1.645,38.332)
GGO/solid	0.041	8.590 (0.967,412.845)
GGO with blood vessels	0.003	5.322 (1.604,21.142)

**Fig. 4:**
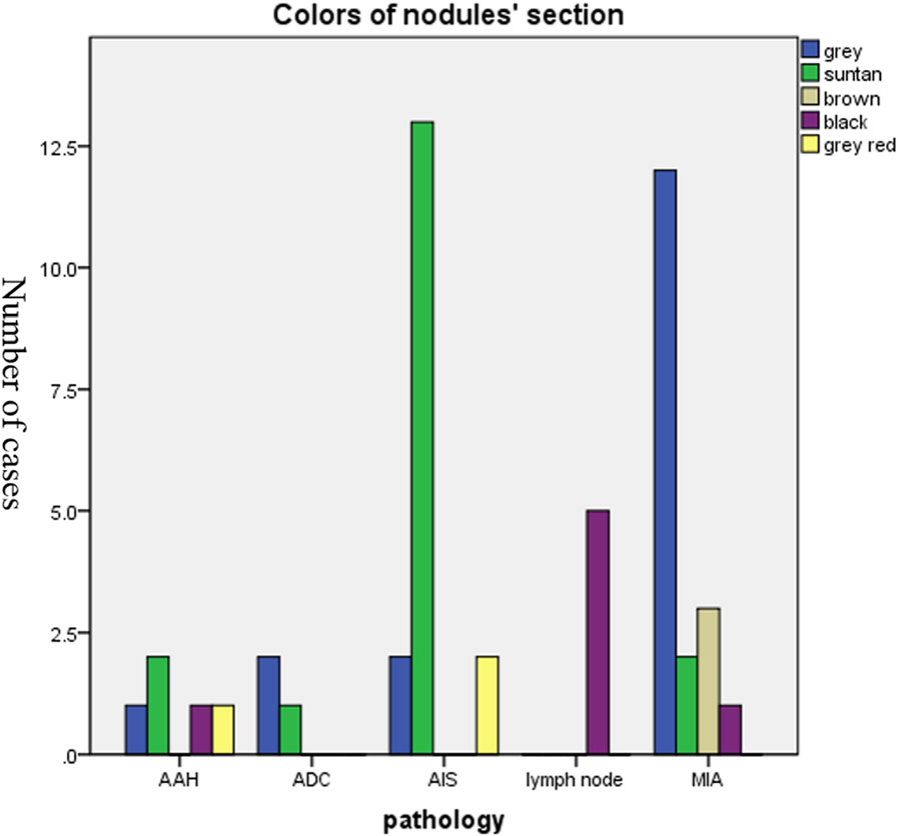
Color distribution of nodule sections in different pathologies

In the present study, the colors of the 66 nodule’s section were also recorded (Fig. 4). In course of AAH, two nodules’ section were suntan and the other three were grey, black and grey red respectively. Among AIS, 76.5% (13/17) of the nodules’ section were suntan. Among MIA, about 66.7% (12/18) of the nodules were grey. All of the lymph node hyperplasia were black.

CT-guided hook-wire implantation was performed without complications in all except in 3 patients (3.7%). Localized pneumothorax and pulmonary hemorrhage were observed in 1 patient with no respiratory symptom, which required no treatment. Two (2.5%) hook wires dislodged before VATS procedure (during patient transport from the interventional radiology suite to the surgical suite), which were most probably due to the relatively shallow of the nodules. Nevertheless, both the two nodules were finally successful resected according to the insertion route of hook wire (by the focal haemorrhagic spot on the visceral pleura). For one dislodgement, the hook wire could not be found either in the pleural cavity or outside the body. We ultimately found it in the chest wall muscles through intraoperative X-ray irradiation. No patient was needed to convert to thoracotomy.

## Discussion

Widespread use of LDCT has led to a great increase in the identification rate of small pulmonary nodules, especially GGO. VATS has been widely utilized in diagnosis and treatment in SPNs since it offers a relatively less traumatic approach to the thoracic cavity, ameliorates visualization of the pleura and the pulmonary surface due to the magnification, and significantly lessen postoperative pain compared with thoracotomy.

Several techniques have been described in the literature for the localization of pulmonary nodules [15–18]. Their disadvantages were also recorded. Methylene blue injection carries the risk of spreading the colorant on the pleural surface, lung parenchyma and the chest cavity during application, which renders subsequent operation difficult, especially in patients with extensive anthracotic pigments. However, Klijian [19] reported that agar mixed with methylene blue injected directly into the SPNs under CT scan were well visualized in entire procedure of operations. The disadvantages of radio-guided localization of SPN are the requirement of radionuclide injection, a magnetic probe connected to a gamma camera, and increased exposure to radiation in the operation. Finger palpation for millimeter nodules during VATS resection is not advisable as small port size and operator dependence make it unreliable. Ultrasonography offers a quick, affordable, less invasive way of localizing lesions but is limited by the presence of air in the lungs [12]. Based on the mentioned above, we compared the advantages and disadvantages between single semi-rigid hook wire, double-thorn hook wire and micro-coil through a swine lung experiment and found that semi-rigid hook wire showed the better operability than the other two, which was already published in December 2017 [13]. In the present study, 74 consecutive patients with 81 pulmonary nodules underwent CT-guided single semi-rigid hook wire localization prior to VATS were examined. The technical success rate was 100%.

Pneumothorax and pulmonary hemorrhage or hematoma are most frequent complications of hook wire placement. Studies have reported the incidence rate of pneumothorax reached to 49, 38%, reported by Junji Ichinose et al. [20] and Matthieu Hanauer et al. [21] respectively. In our study, pneumothorax was observed only in 3 patients (3.7%) immediately after the procedure, which was lower than previous reports. There is also study that is consistent with our conclusions, with an incidence rate of 2% [22]. However, the incidence of pneumothorax is likely to be underestimated. These pneumothoraces occurred in our situation were minimal and do not need any intervention or treatment (for example aspiration or chest tube insertion). Pulmonary hematoma was occasionally reported after hook wire insertion (2/81, 2.5%). Matthieu Hanauer and colleges also reported of a relatively lower incidence (7.2%) of pneumothorax. Since most nodules locate at the periphery of the lung, hematoma were most likely due to the parenchyma bleeding [21]. Other morbidities, including hemoptysis and VAE (venous air embolism, which is a life-threatening complication occasionally reported after hook wire insertion), was not observed in our series. The technique was well tolerated by all the patients.

Dislodgement has been considered a problem of hook wire localization [12, 20, 23, 24], especially in conventional rigid hook wire [25]. Two dislodgement occurred prior to VATS in transferring the patients from positioning room to operating theatre, with a rate of 2.5%. Both the two nodules are GGO. The distance from the nodule to pleura surface of the two cases are 8 mm and 7 mm, which we consider that too close to the pleura be the probably reason for dislodgement. With this condition, we could choose to insert the wire a little deeper than the nodule to avoid dislodgement in later cases. Dendo et al. [24] and Xuanli Xu et al. [26] both reported a dislodgement rate of 2.4%, which is consistent with our findings. However, a recent systematic review reported by Chul Hwan Park and his colleges hold the point that hook-wire localization had a relatively lower successful targeting rate than microcoil and lipiodol localization because of dislodgement or migration, with a failure rate 6.1% [27]. Thus, dislodgement remains a major limitation of this technique. However another study [20] contained 417 localizations reported a dislodgement rate of only 0.4%. The dislodgements occurred in our situation probably because of the depth of nodule is shallow.

Generally, hook wire procedure does not take much time. In our series, the mean time of the localization procedure were 10 min. Additionally, the vicinity of radiology unit and the operating room makes it easy to transport patients. The mean delay was half an hour between the localization and operation. Due to the flexibility of the location system, patients did not complain about pleural pain accompanying breathing during and after localization. Our rate of dislodgement was acceptable and the technique was well tolerated by the patients.

In the present study, 74 patients with 81 SPNs were successfully resected by VATS. We found that SPNs were more frequently detected in female, with a sex ratio of 42:32 (female: male), and lung cancer incidence was higher in women than in men, which probably because of the increasing incidence of lung cancer in women [28]. An important finding in the present study was that pGGO were mostly diagnosed as AIS, MIA and AAH, and solid nodules had a much higher probability of benign, with a rate of 85.7%, which is consistent with previous studies [8, 9]. Whereas study reported by Xuanli Xu [26] showed pure GGOs were more likely diagnosed as AAH. For mGGOs, they were more likely to be malignant, with a rate of 57.9%. We also found that approximately one fourths of pure GGOs were AIS. More than half of mGGOs were diagnosed as lung cancer. In our series, we carefully recorded the external characteristics of GGOs, an important finding showed that GGOs with tiny blood vessels was statistically correlated with malignancy (OR, 5.322, 95%CI. 1.604, 21.142), which would be helpful for thoracic surgeon to make more accurate judgement of nodules. Lung cancer tended to develop in older patients with larger nodule diameters, and the majority patients with SPN were asymptomatic. A multivariate analysis [21] has identified that nodules with a size more than 10 mm (OR: 3.61) were related to malignancy, which is similar to our study. In our series, no significant difference was found in nodule localization (upper or lower lobe) between malignant and benign, whereas Xuanli Xu et.al and Matthieu Hanauer et.al reported nodules localized in upper lobe were more frequently malignant (OR: 3.61).

The colors of the nodules section were simultaneously identified. We found that the color of AIS and MIA were mostly suntan and grey respectively. The lymph node hyperplasia is generally black. Knowing this can help us pre-diagnose the tumor intraoperative, from which we can reduce the duration of the surgery and the complications caused by long operation time.

## Conclusion

VATS resection after CT-guided semi-rigid single hook wire localization for SPN is practical and safe that allows for proper diagnosis and treatment with a high success rate. SPN (especially GGO) larger than 10 mm is commonly diagnosed as malignancy compared with the one less than 10 mm, such SPN requires to be taken seriously. Additionally, the incidence of lung cancer may be younger than previously reported since the screening with low-dose CT.

## Data Availability

The data in this manuscript can be found in Xiamen University the first affiliated hospital.

## Abbreviations

CI: Confidence interval
CT: Computed tomography
GGO: Ground-glass opacity
SPN: Small pulmonary nodule
VATS: Video-assisted thoracoscopic surgery
